# Functional and phylogenetic analysis of TetX variants to design a new classification system

**DOI:** 10.1038/s42003-022-03465-y

**Published:** 2022-05-31

**Authors:** Qipeng Cheng, Yanchu Cheung, Chenyu Liu, Edward Wai Chi Chan, Kwok Yin Wong, Rong Zhang, Sheng Chen

**Affiliations:** 1 State Key Lab of Chemical Biology and Drug Discovery, Department of Applied Biology and Chemical Technology, The Hong Kong Polytechnic University, Hung Hom, Kowloon Hong Kong; 2 Department of Infectious Diseases and Public Health, Jockey Club College of Veterinary Medicine and Life Sciences, City University of Hong Kong, Kowloon, Hong Kong; 3 Department of Clinical Laboratory, Second Affiliated Hospital of Zhejiang University, School of Medicine, Hangzhou, 310009 People’s Republic of China

**Keywords:** Antimicrobial resistance, Bacterial evolution

## Abstract

Recently, many TetX variants such as Tet(X3~14) were reported to confer resistance to tigecycline which is a last-resort antibiotic used to treat infections caused by multidrug-resistant bacteria. In this study, we identified essential residues including 329, 339, 340, 350, and 351 in TetX variants that mediated the evolution of the tigecycline-inactive Tet(X2) enzyme to the active forms of Tet(X3) and Tet(X4). Based on their amino acid sequences and functional features, we classified TetX variants into TetX-A class, TetX-B class and TetX-C class. We further found that TetX-A class variants originated from Bacteroidetes, with some variants further evolving to TetX-C class and acquired by Enterobacteriaceae. On the other hand, our data showed that some variants genes belonging to TetX-A class evolved directly to TetX-B class, which was further transmitted to *Acinetobacter* spp. This new classification system may facilitate better clinical management of patients infected by TetX-producing strains.

## Abstract (web summary)

TetX variants such as Tet(X3~14) were reported to confer resistance to tigecycline which is a last-resort antibiotic used to treat infections caused by multidrug-resistant bacteria. Here, Cheng et al. propose a new classification system for TetX variants.

## Introduction

Tetracyclines are a group of antibiotic compounds that have a common basic structure (a linear fused tetracyclic nucleus) which exhibit activity against a wide range of microorganisms, including Gram-positive and Gram-negative bacteria^1^. Because of their broad-spectrum activity and the low cost, tetracyclines are extensively used in clinical treatment of human infections, as well as applications in the field of veterinary medicine and agriculture, since their discovery in the 1940s. Tetracyclines inhibit protein synthesis by binding reversibly to the bacterial 30 S ribosomal subunit and arresting translation, exerting steric hindrance effect on the docking of aminoacyl-transfer RNA (tRNA) during elongation^2^. Due to the widespread use of tetracyclines, resistance in clinical and food isolates is increasingly reported. A range of mechanisms of resistance are known to mediate tetracycline resistance, such as efflux pumps, ribosomal protection, rRNA mutations and enzymatic inactivation^3^. To counteract these resistance mechanisms, a semisynthetic glycylcycline known as tigecycline was approved for clinical use by FDA in 2005^4^. Tigecycline has become increasingly important in treating bacterial infections, since it is one of the few antibiotics which is still effective against a range of newly emerged multidrug-resistant Gram-positive and Gram-negative bacterial strains^5^. Although tigecycline can overcome two main resistance mechanisms, namely ribosomal protection and efflux, resistance to tigecycline has been reported^3,6,7^. In particular, several plasmid-borne *tet*(X) variant genes that confer clinically significant level of tigecycline resistance have recently been detectable among clinical strains, compromising the effectiveness of this relatively new tetracycline drug in clinical treatment of bacterial infection^8–12^.

**Fig. 1:**
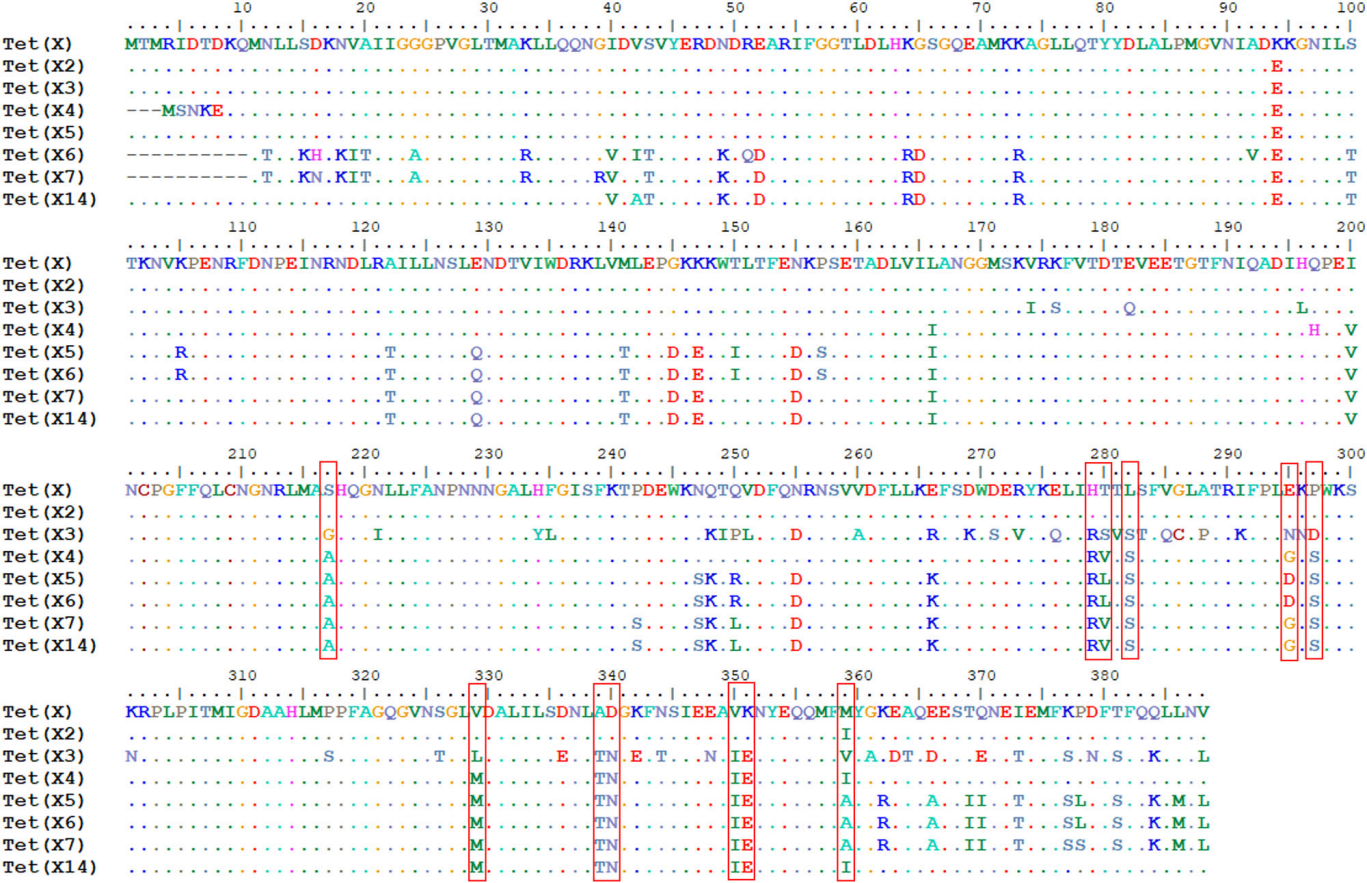
Alignment of the complete amino acid sequences encoded by *Tet*(X) variants genes using BioEdit. Amino acid residues are depicted in different color, the same amino acid is shown as dots in the alignment. Commonly mutated sites in Tet(X3), Tet(X4), Tet(X5), Tet(X6), and Tet(X7) compared with original TetX protein are highlighted in red box.

TetX is one of the flagship tetracycline-inactivating enzymes that can catalyze the degradation of tetracyclines, which was first proposed as a resistance mechanism in 1984^13^. As a flavin-dependent monooxygenase, flavin adenine dinucleotide (FAD) as a cofactor bound to TetX and TetX strictly required exogenous nicotinamide adenine dinucleotide phosphate (NADPH) to catalyze the oxidation reaction which inactivates most of the tetracyclines in vitro, including tigecycline^14,15^. To date, several TetX variants, designated as, TetX, Tet(X1), Tet(X2), Tet(X3), Tet(X4), Tet(X5), Tet(X6), Tet(X7), Tet(X8), Tet(X9), Tet(X10), Tet(X11), Tet(X12), Tet(X13), and Tet(X14) have been identified in various bacterial species. Compared to TetX, Tet(X2) only has one mutation at residue 94 (Fig. 1). Tet(X1) and Tet(X2) exhibit 66.8% and 99.4% amino acid identities with the original TetX, respectively. While the variant Tet(X1) is a truncated protein that lacked the FAD-binding domain and has been proven to be unable to catalyze degradation of tetracyclines, Tet(X2) exhibits degradative activity towards tigecycline. Tet(X3), Tet(X4), Tet(X5), Tet(X6), Tet(X7), and Tet(X14) were identified in *Acinetobacter, Escherichia coli*, *Myroides phaeus*, *Proteus spp*., *Pseudomonas aeruginosa*, and *Empedobacter stercoris* and found to exhibit 85.5%, 95.4%, 89.6%, 84.3%, 85.4%, and 90.7% amino acid identities with the original TetX, respectively^8–12,16–18^. These variants confer high-level resistance to tigecycline (8–32 mg/L). Based on these findings, we hypothesize that an increasing number of TetX variants that can confer tigecycline-resistance will continue to emerge as a result of deep surveillance of clinical and food isolates. Designating the TetX variants an increment number is not an appropriate way to label a large number of functionally different enzyme variants. It is necessary to design a new system for functional classification for TetX variants to facilitate clinical management of infections caused by bacterial strains that produced different TetX variants, and therefore exhibit different levels of susceptibility to tigecycline. In this study, we propose that TetX variants should be classified into three major groups, namely TetX-A class, TetX-B class, and TetX-C class, depending on enzymatic activity and genetic features.

## Results

### Identification of key residues that contributed to elevated tigecycline MICs of TetX variants

**Fig. 2:**
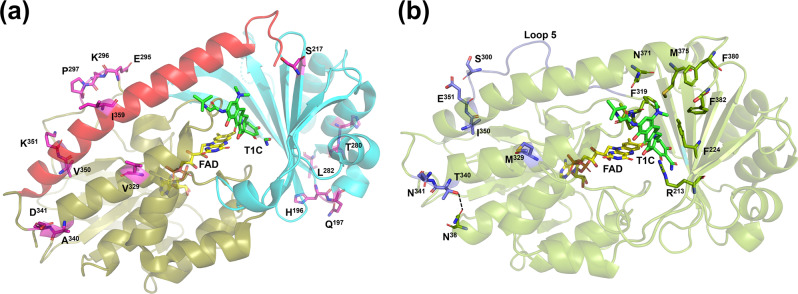
Location of related residues in the Tet(X)-tigecycline complex structure. **a** Mapping the test amino acid substitution sites in the FAD-binding domain (deep olive), substrate-binding domain (cyan), and C-terminal helix (red). Residues are depicted as pink stick. **b** FAD and substrate-binding sites and mutation residues are showed in the model of Tet(X2) mutant (V^329^M/A^339^T/D^340^N/V^350^I/K^351^E). Loop 5 and mutant residues are shown as deep blue. Substrate-binding sites are depicted as green stick.

Tet(X3), Tet(X4), Tet(X5), Tet(X6), Tet(X7), Tet(X14) but not TetX and Tet(X2), were shown to confer resistance to tigecycline. We aligned the representative amino acid sequences of each of these enzymes in an attempt to identify common amino acid substitutions in Tet(X3) to Tet(X14) that might contribute to elevated tigecycline MIC. Several common changes at residues S^217^, H^279^, T^280^, L^282^, E^295^, P^297^, V^329^, A^339^, D^340^, V^350^, K^351^ and I^359^ were found (Figs. 1 and 2). When compared to Tet(X2), Tet(X4) exhibited a smaller number of changes than Tet(X3), Tet(X5), Tet(X6), Tet(X7), and Tet(X14). Since Tet(X4)-producing strains are resistant to tigecycline, it is likely that the amino acid sequence variations between Tet(X2) and Tet(X4) are responsible for the elevated tigecycline MIC of Tet(X4)-producing strains and are therefore the focus of our mutation analysis (Fig. 1). We then tested the effect of single amino acid substitution in these residues using Tet(X2) as template. Our data showed that each of the H^196^L, Q^197^H, S^217^G, H^279^R, T^280^L, E^295^G, E^295^N, E^295^D, K^296^N, P^297^D, P^297^S, D^340^N, V^350^I, I^359^M, I^359^V changes exhibited little effect on the MIC of tigecycline by itself, whereas each of the S^217^A, T^280^V, T^280^S, L^282^S, E^295^N, V^329^L, V^329^M, A^339^T, and K^351^E changes alone contributed slightly to tigecycline resistance. In particular, strains carrying the L^282^S substitution exhibited 4-fold increase of MIC when compared to Tet(X2)-producing strains (Table 1). Mutants harboring double and multiple substitutions were further created and tested, with results showing that the A^339^T/D^340^N, and V^350^I/K^351^E double mutants exhibited 4-fold increase in MIC when compared to strains producing Tet(X2). Strains that contain amino acid substitutions at three sites, such as those carrying the V^329^L/ A^339^T/D^340^N, V^329^L/ V^350^I/K^351^E, V^329^M/ A^339^T/D^340^N, and V^329^M/ V^350^I/K^351^E changes, exhibited tigecycline MIC of 8, 4, 4, 8 mg/L, which represent 8, 4, 4 and 8-fold increase, respectively. Multiple mutations, such as those which lead to as many as four amino acid changes (A^339^T/D^340^N/V^350^I/K^351^E), also caused the tigecycline MIC to increase to 8 mg/L. Furthermore, two mutants which contained five amino acid changes, namely V^329^L/A^339^T/D^340^N/V^350^I/K^351^E and V^329^M/A^339^T/D^340^N/V^350^I/K^351^E, both exhibited tigecycline MIC of 16 mg/L, which is similar to that of Tet(X3) and Tet(X4) (Table 1). This mutation analysis therefore allowed us to identify important residues that mediated the evolution of Tet(X2) to Tet(X3) and Tet(X4). On the other hand, we made two reverse penta mutants at position 329, 339, 340, 350, and 351 for Tet(X3) and Tet(X4), respectively. Tet(X3)-L^329^V/T^339^A/N^340^D/I^350^V/E^351^K and Tet(X4)-M^329^V/T^339^A/N^340^D/I^350^V/E^351^K exhibited decreasing MIC (2, 2 mg/L) against tigecycline comparing to the wild type, respectively (Table 1). It was also supported that these five specific substitutions at Tet(X3) and Tet(X4) were important for tigecycline resistance. It should be noted that the mutants created in this work which exhibited higher MICs to tigecycline also exhibited elevated MICs of other tetracycline antibiotics, such as tetracycline, and minocycline (Table 1).

**Table 1:** MICs for *E. coli* BW25113 strains harboring a pBAD18 plasmid which contains a *tet*(X2), *tet*(X3), *tet*(X4), mutated *tet*(X2) gene, mutated *tet*(X3) gene or mutated *tet*(X4) gene.

*E. coli* Strains	MIC (mg/L)		
	TGC	MIN	TET^a^
*E. coli* 25922	0.25	0.25	1
Vector control	0.5	1	2
*tet*(X2)	1	4	16
*tet*(X3)	16	16	128
*tet*(X4)	16	16	128
H^196^L^b^	1	4	32
Q^197^H	1	2	32
S^217^A	2	4	32
S^217^G	1	2	16
H^279^R	1	4	16
T^280^V	2	4	16
T^280^L	1	4	16
T^280^S	2	4	16
L^282^S	4	8	32
E^295^G	1	4	32
E^295^N	2	4	32
E^295^D	1	4	16
K^296^N	1	2	16
P^297^D	1	2	8
P^297^S	1	4	16
I^359^M	1	4	16
I^359^V	1	4	32
V^329^L	2	8	32
V^329^M	2	8	32
A^339^T	2	4	32
D^340^N	1	4	32
V^350^I	1	4	32
K^351^E	2	4	32
A^339T^/D^340^N	4	8	64
V^350I^/K^351^E	4	8	64
V^329^L/A^339T^/D^340^N	8	8	64
V^329^L/V^350I^/K^351^E	4	8	64
V^329^M/A^339T^/D^340^N	4	8	128
V^329^M/V^350I^/K^351^E	8	16	64
A^339T^/D^340^N/V^350I^/K^351^E	8	8	64
V^329^L/A^339^T/D^340^N/V^350^I/K^351^E	16	16	128
V^329^M/A^339^T/D^340^N/V^350^I/K^351^E	16	16	64
*tet*(X3)-L^329^V/T^339^A/N^340^D/I^350^V/E^351^K^c^	2	8	64
*tet*(X4)-M^329^V/T^339^A/N^340^D/I^350^V/E^351^K^d^	2	8	64

^a^*TET* tetracycline, *MIN* minocycline, *TGC* tigecycline.

^b^All mutants are derived from pBAD18-*Tet*(X2) by site-directed mutagenesis.

^c^*tet*(X3)-L^329^V/T^339^A/N^340^D/I^350^V/E^351^K is reverse penta mutant derived from pBAD18-*tet*(X3).

^d^*tet*(X4)-M^329^V/T^339^A/N^340^D/I^350^V/E^351^K is reverse penta mutant derived from pBAD18-*tet*(X4).

To have a biochemical correlate to the MIC data, constructed mutants with boosted MICs were purified and steady-state kinetic parameters of these protein were also determined for tigecycline (Table 2 and Supplementary Fig. 1). Firstly, we found the catalytic efficiency of Tet(X3) and Tet(X4) was about 1.2–4.8 folds greater than that of Tet(X2) for hydrolysis of tigecycline, *k*_cat_ */K*_*m*_ values of them are 8.09 × 10^5 ^M^−1^ S^−1^, 1.16 × 10^6 ^M^−1^ S^−1^ and 2.33 × 10^5 ^M^−1^ S^−1^, respectively (Table 2). The most mutants showed higher catalytic efficiencies on tigecycline than that of Tet(X2), which is agreed with the results of increased MICs. The single amino acid change mutants such as S^217^A, T^280^V, T^280^S, L^282^S, E^295^N, and V^329^L did not increase 2-fold changes in catalytic efficiencies on tigecycline. In contrast, 3.5-fold, 2.2-fold and 8.4-fold increases were observed for the V^329^M, A^339^T, and K^351^E mutants, respectively. Consistent with the MIC data, multiple mutants V^350^I/K^351^E, V^329^L/A^339^T/D^340^N, V^329^L/V^350^I/K^351^E, V^329^M/A^339^T/D^340^N, V^329^M/V^350^I/K^351^E, A^339^T/D^340^N/V^350^I/K^351^E, V^329^L/A^339^T/D^340^N/V^350^I/K^351^E and V^329^M/A^339^T/D^340^N/V^350^I/K^351^E exhibited more than 2-fold increase in catalytic efficiency for tigecycline hydrolysis (Table 2). Except for A^339^T/D^340^N, which displayed slightly change in catalytic efficiency. While the catalytic efficiency of penta mutant Tet(X3)-L^329^V/T^339^A/N^340^D/I^350^V/E^351^K and Tet(X4)-M^329^V/T^339^A/N^340^D/I^350^V/E^351^K was lower than that of Tet(X3) and Tet(X4) but was about 1.3–3.1 folds higher than that of Tet(X2) for hydrolysis of tigecycline (Table 2), which correlated well to their MICs. In summary, acquisition of these single and multiple substitutions associated with the variants allows Tet(X2) to hydrolyze tigecycline more efficiently.

**Table 2:** Kinetic parameters (± SD) of the TetX proteins on tigecycline.

Protein	*k*_cat_ (S^−1^)	*K*_m_ (µM)	*k*_cat_/K_m_ (M^−1^ S^−1^)
Tet(X2)	1.04 ± 0.01	4.45 ± 0.13	2.33 × 10^5^
Tet(X3)	2.16 ± 0.05	2.97 ± 0.2	8.09 × 10^5^
Tet(X4)	2.00 ± 0.02	1.73 ± 0.09	1.16 × 10^6^
S^217^A	2.13 ± 0.07	4.58 ± 0.41	4.65 × 10^5^
T^280^V	0.76 ± 0.01	3.02 ± 0.16	2.51 × 10^5^
T^280^S	1.15 ± 0.03	3.09 ± 0.24	3.72 × 10^5^
L^282^S	1.36 ± 0.05	3.96 ± 0.38	3.43 × 10^5^
E^295^N	1.39 ± 0.07	4.92 ± 0.64	2.83 × 10^5^
V^329^L	0.97 ± 0.03	3.56 ± 0.35	2.72 × 10^5^
V^329^M	3.80 ± 0.11	5.18 ± 0.34	7.34 × 10^5^
A^339^T	1.95 ± 0.11	3.69 ± 0.50	5.28 × 10^5^
K^351^E	6.82 ± 0.40	3.48 ± 0.47	1.96 × 10^6^
A^339^T/D^340^N	0.95 ± 0.05	3.81 ± 0.49	2.49 × 10^5^
V^350^K/I^351^E	1.64 ± 0.01	3.51 ± 0.54	4.67 × 10^5^
V^329^L/A^339^T/D^340^N	1.29 ± 0.03	1.05 ± 0.08	1.23 × 10^6^
V^329^L/V^350^I/K^351^E	1.13 ± 0.03	2.22 ± 0.17	5.09 × 10^5^
V^329^M/A^339^T/D^340^N	3.94 ± 0.14	4.25 ± 0.39	9.27 × 10^5^
V^329^M/V^350^I/K^351^E	1.99 ± 0.06	2.81 ± 0.24	7.08 × 10^5^
A^339^T/D^340^N/V^350^I/K^351^E	1.69 ± 0.06	2.89 ± 0.30	5.85 × 10^5^
V^329^L/A^339^T/D^340^N/V^350^I/K^351^E	1.38 ± 0.03	2.03 ± 0.13	6.79 × 10^5^
V^329^M/A^339^T/D^340^N/V^350^I/K^351^E	1.95 ± 0.13	3.53 ± 0.52	5.52 × 10^5^
Tet(X3)-L^329^V/T^339^A/N^340^D/I^350^V/E^351^K	0.82 ± 0.02	2.65 ± 0.29	3.09 × 10^5^
Tet(X4)-L^329^V/T^339^A/N^340^D/I^350^V/E^351^K	2.89 ± 0.13	3.95 ± 0.61	7.31 × 10^5^

In addition, the expression levels of Tet(X2), Tet(X3), Tet(X4) and mutants were also detected by Western Blotting. All test proteins displayed small changes (0.8–1.5 folds) comparing to Tet(X2) under T7 promoter in *E. coli* BL21(DE3) (Supplementary Fig. 2). It was indicated that Tet(X3), Tet(X4) and mutants with increasing MICs showed high resistance to tigecycline is the result of their catalytic efficiencies and is not likely to be due to production of higher amount of protein.

### Mapping amino acid substitutions in the TetXs’ structures

To investigate how these amino acid substitutions mediated changes in Tet(X2) activity, we mapped the site of these substitutions against the complex structure of Tet(X2) with tigecycline (Fig. 2a) and found that the H^196^L, Q^197^H, S^217^A, H^279^R, T^280^S, T^280^V, T^280^L and L^282^S changes, which are commonly found in Tet(X3) to Tet(X14), occur within the second domain of the protein (cyan), which is implicated largely in tigecycline recognition. On the other hand, the other commonly found changes in the high activity variants of tetracycline, such as E^295^D, E^295^G, E^295^N, K^296^N, P^297^D, P^297^S, V^329^M, A^339^T, and D^340^N changes, were found to occur in the FAD-binding domain (deep olive). In addition, residues where the V^350^I, K^351^E, I^359^M and I^359^V occur were located in a C-terminal alpha-helix (red), which could stabilize the other two domains^19^. The T^280^ residue was closer to the putative O_2_ binding pockets and has previously been suggested to interfere with O_2_ diffusion. Therefore, the T^280^V or T^280^S change might also affect O_2_ transport within the enzyme. In previous directed evolution studies, a mutant carrying the T^280^A change could be selected in the presence of minocycline and tigecycline^20,21^. The structure of the Tet(X2) (T^280^A) complex with minocycline showed that position 280 was not directly involved in the catalytic mechanism of the enzyme but the T^280^A substitution Tet(X2) (T^280^A) changed the enzyme kinetics of Tet(X2) indirectly perhaps through altered the protein dynamics^20^. In the Tet(X2) (V^329^M/A^339^T/D^340^N/V^350^I/K^351^E) mutant model, the A^339^T substitution makes van der Waals contact with the side chain of N^38^ (2.9–3.8 Å, Fig. 2b) and may stabilize the link between the α helix 11 and α helix 1. Stabilization of α helix 11, which was involved in FAD binding and tigecycline recognition, could increase the catalytic activity of Tet(X2) (Fig. 2b). In addition, when lysine was substituted by glutamic acid at position 351, the electrostatic potential in the area between Loop 5 and C-terminal α helix became more negatively charged and the bridge between E^351^ and S^300^ was broken (Supplementary Fig. 3). The change in electrostatic potential may influence substrate binding because residues N^371^, M^375^, F^380^, F^382^ in the C-terminal α helix also interacts with tigecycline (Fig. 2b). Structural analysis showed that the reason why these mutational changes can mediate evolution from Tet(X2) to Tet(X3) and Tet(X4) is that they result in stabilization of the α helixes that are part of the active site, thereby fine tuning the active site conformation to allow better substrate recognition, rather than directly exerting an impact on recognition and binding of tigecycline.

### Classification of TetX variants

**Fig. 3:**
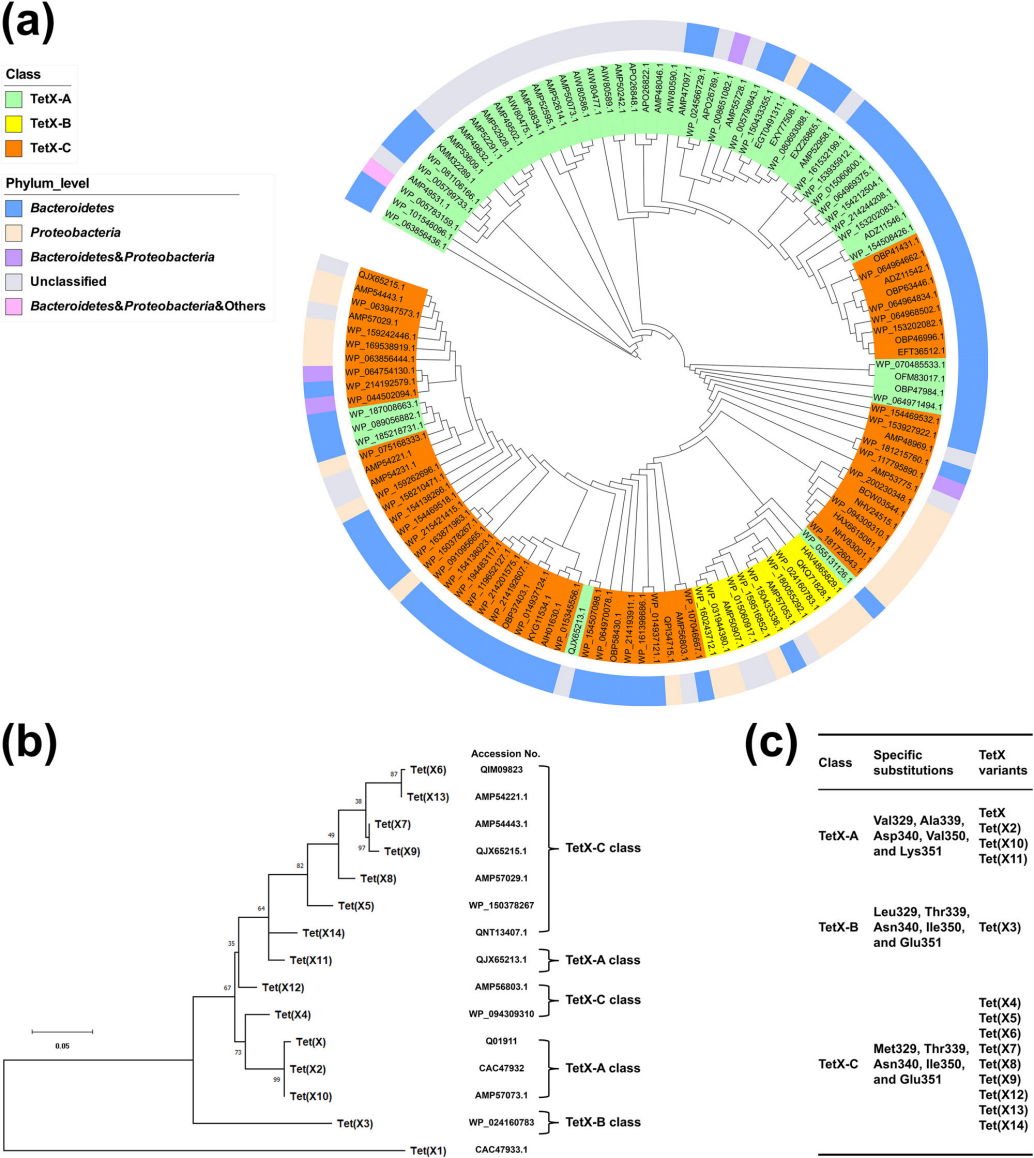
Phylogenetic analysis of TetX like protein. **a** TetX-related proteins are divided into three classes: TetX-A (green), TetX-B (yellow), and TetX-C (orange). Phylogeny is inferred by using the maximum-likelihood method and Flu +G + I model. A discrete Gamma distribution approach was used to depict the difference in evolutionary rate among the sites [4 categories (Gamma shape parameter = 0.609)]. This analysis involved 128 amino acid sequences. Different species hits the protein sequences organized in the phylum level. *Bacteroidetes*, *Proteobacteria*, *Firmicutes*, *Spirochetes*, *Bacteroidetes* plus *Proteobacteria*, and unclassified organisms are shown in blue, pink, light purple, and gray, respectively. Evolutionary analyses were conducted in online PlyML 3.0. The tree was visualized using iTOL (ITEREACTIVE TREE OF LIFE). **b** Phylogenetic analysis of the amino acid sequences of the reported TetX variants. The maximum-likelihood tree was inferred using MEGA X^35^. The tree is drawn to scale, with branch lengths measured in the number of substitutions per site. **c** Reported TetX variants are distributed in TetX-A class, TetX-B class, and TetX-C class with specific substitutions.

A PST-BLAST search with a TetX variant (Accession No: WP_063856436.1) as the template sequence returned a total of 128 related homologs with the annotation of TetX. Based on the analysis of the multiple sequence alignment, we defined that sequences lacking the A^339^T/D^340^N and V^350^I/K^351^E changes always belonged to TetX-A class, which is consistent with previous classification^15^. Two variants were originally defined as Tet(X10) and Tet(X11) could be classified into new TetX-A class (Fig. 3)^12^. Here, protein sequence with the V^329^M substitution, A^339^T/D^340^N, and V^350^I/K^351^E changes, should be regarded as TetX-C class. These enzyme variants were originally defined as Tet(X4), Tet(X5), Tet(X6), Tet(X7), and Tet(X14) (Fig. 3)^8–12,16–18^. Our new definition is based on their close sequence homology with Tet(X2) and similar activity on tigecycline according to our mutational analysis data, which showed that the V^329^M, A^339^T/D^340^N, and V^350^I/K^351^E amino acid substitutions elevated tigecycline MICs to the same level as original Tet(X4), Tet(X5), Tet(X6) and Tet(X7) (Table 1). Another class comprises enzymes which contain the A^339^T/D^340^N, V^350^I/K^351^E and V^329^L changes; these enzymes were originally defined as Tet(X3). We also propose that they should be classified as TetX-B class. Two independently reported TetX variants from *Acinetobacter baumannii* and *Empedobacter brevis* could be classified into this class^8,22^. This definition is based on the fact that it contains one different substitution at position 329 when compared to TetX-C defined above. Consistently, carriage of similar patterns of important amino acid substitutions including V^329^L, A^339^T/D^340^N, V^350^I/K^351^E, implied that the catalytic activity of the newly defined TetX-B enzyme is similar to TetX-C class and played a key role in elevating tigecycline MICs. Based on the effect of these amino acid changes in enzyme functions, there might be two branches of the evolution pathway for TetX variants. Apart from the one which involves evolution from TetX-A to the newly defined TetX-B and TetX-C, respectively (Fig. 3). To conclude, based on functional characterization and sequence alignment, we propose to classify TetX variants into three classes, namely TetX-A class, TetX-B class and TetX-C class.

Furthermore, a polygenetic tree was constructed for the 128 annotated TetX variants. Our data showed that the phylogenetic tree was highly consistent with the functional classification scheme we proposed (Fig. 3a). The TetX variant (WP_063856436.1) from *Bacteroides fragilis* was used to root the phylogenetic tree, tree was divided into four substitution rate categories. Four major branches can be depicted by the phylogenetic tree and are aligned well to our newly defined TetX-A class, TetX-B class, and TetX-C class. TetX variants evolved originally from the TetX-A class to TetX-C class, and subsequently to TetX-B. It should be noted, however, that categorization of some TetX variants by functional classification is not consistent with their position in phylogenetic analysis. For example, three variants belonging the TetX-A class (WP_187008663.1, WP_089056882.1, and WP_185218731.1) were under a same phylogenetic branch with TetX-C class protein (Fig. 3). Protein sequence alignment showed that these three variants exhibited a high degree of identity (91-94%) with the TetX variant (WP_075168333.1) of TetX-C class (Supplementary Fig. 4), whereas there are differences for the essential residues at position 329, 339, 340, 350, and 351. Therefore, functionally, these three sequences should belong to TetX-A. It is possible that these three sequences could be the progenitor sequences that evolved from TetX-A into TetX-C in that branch.

### Bacterial species specificity analysis of different TetX variants

Further analysis of the BLAST results showed that these 128 annotated TetX variants were harbored by 497 bacterial strains of various species, among which 414 strains belonged to Bacteroidetes and Proteobacteria, another two strains are from Firmicutes and Spirochetes, respectively, the rest could not be classified (Fig. 3 and Table 3). The newly defined TetX-A class enzymes were almost exclusively produced by Bacteroidetes, suggesting that this gene originated from Bacteroides. This conclusion is consistent with those of previous reports^23^. Six TetX-A class variants could be detected in Proteobacteria, suggesting that these genes were subsequently transmitted from Bacteroides to Proteobacteria. The newly defined TetX-C class variants are also commonly carried by Bacteroides, with *Riemerella spp*. being the most commonly species, suggesting that TetX-C variants might originate from this bacterial species. In addition, TetX-C class variants were shown to be highly prevalent among members of Proteobacteria including Enterobacterales, and Pseudomonadales. In contrast, TetX-B class was mainly reported in *Acinetobacter spp*., with two being reported in *Empedobacter brevis* (Table 3).

**Table 3:** Distribution and relative prevalence of newly defined *tet*X-A, *tet*X-B and *tet*X-C genes among different bacterial species.

Phylum	Order	Family	Species	TetX-A	TetX-B	TetX-C
Bacteroidetes	Bacteroidales	Rikenellaceae	*Alistipes*	3		
		Bacteroidaceae	*Bacteroides*	32		5
			*Phocaeicola vulgatus*	1		
		Odoribacteraceae	*Odoribacter spp*.	2		
			*Butyricimonas paravirosa*	1		
		Porphyromonadaceae	*Parabacteroides*	7		
		Prevotellaceae	*Prevotella copri*	1		
		Other	*Bacteroidales bacterium*	4		1
	Chitinophagales		*Chitinophagales bacterium*	1		
			*Capnocytophaga spp*.			1
		Flavobacteriaceae	*Chryseobacterium spp*.	10		3
			*Flavobacterium spp*.	5		2
			*Myroides spp*.	6		1
			*Elizabethkingia spp*.	3		
			*Empedobacter brevis*	3	2	
	Flavobacteriales	Weeksellaceae	*Empedobacter stercoris*	2		1
			*Empedobacter falsenii*	2		
			*Ornithobacterium*	1		
			*Riemerella spp*.	19		83
			*Weeksella spp*.	2		1
			*Sphingobacterium spp*.	6		
	Sphingobacteriales	Sphingobacteriaceae	*Parapedobacter spp*.			2
			*Pedobacter spp*.	2		
	Ignavibacteriales	Ignavibacteriaceae	*Ignavibacteria bacterium*	1		
	Other		*Bacteroidetes bacterium*	1		
	Aeromonadales	Aeromonadaceae	*Aeromonas caviae*			1
Proteobacteria	Alteromonadales	Shewanellaceae	*Shewanella algae*			1
	Burkholderiales	Comamonadaceae	*Delftia.sp*	1		
	Campylobacterales	Campylobacteraceae	*Campylobacter coli*			1
	Enterobacterales	Enterobacteriaceae	*Citrobacter spp*.			2
			*Escherichia spp*.	1		92
			*Enterobacter hormaechei*			
			*Enterobacteriaceae bacterium*	1		
			*Klebsiella spp*.			4
			*Salmonella spp*.			12
			*Shigella sonnei*			1
		Morganellaceae	*Proteus*			13
			*Providencia*	2		1
	Pseudomonadales	Moraxellaceae	*Acinetobacter spp*.		42	17
		Pseudomonadaceae	*Pseudomonas aeruginosa*			2
	Vibrionales	Vibrionaceae	*Vibrio cholerae*	1		
	Other		*Gammaproteobacteria bacterium*			2
Firmicutes	Lactobacillales	Streptococcaceae	*Streptococcus gordonii*	1		
Spirochetes	Spirochaetales	Spirochaetaceae	*Treponema spp*.	1		
Unclassified				47	16	18
Total				170	60	267

## Discussion

Tet(X/X2) is known to confer resistance to tetracycline. Its significance was brought up recently due to the reports of new variants of Tet(X2), namely Tet(X3~14), which are responsible for causing resistance to tigecycline among members of Enterobacteriaceae, as tigecycline has become the last-resort antibiotic to treat clinical infections caused by Carbapenem-resistant Enterobacteriaceae (CRE). The increasing prevalence of clinical strains producing these TetX variants will undermine the choice of treatment for clinical CRE infections. However, due to presence of various TetX-related amino acid sequences available in GenBank and a lack of data regarding the functional characteristic of these protein sequences, the current definition of TetX variants is confusing. With the advent of sequencing technology in recent years, the number of sequences of TetX variants deposited into the Genbank will continue to increase, rendering current nomenclature of TetX variants insufficient to depict the functional types of these enzymes. It is urgent to develop a new classification system according to the functional and amino acid sequence characteristics of TetX variants. The key problem in developing a classification system for TetX variants lies in the large sequence variation between TetX variants. We believe that the best classification system for TetX variants should be a function-based system. In this study, we tested this logic and identified functionally amino acid substitutions that can help distinguish between Tet(X/X2) which exhibit no or very low catalytic activity on tigecycline, and TetX variants with high catalytic activity. Using sequence comparison and mutational analysis, we identified key residues that enable us to classify some TetX variants as a new TetX-C class. With the signature amino acid substitutions of V^329^M/A^339^T/D^340^N/V^350^I/K^351^E, most of these newly defined TetX-C variants were originally being named as Tet(X4), Tet(X5), Tet(X6), Tet(X7), and Tet(X14). From the evolutionary viewpoint and the perspective of phylogenetic relationship, these variants exhibit the closest genetic relationship with TetX-A class and should therefore classified as TetX-C class. The newly TetX-B class comprise most of the original Tet(X3) enzymes; apart from the V^329^L/A^339^T/D^340^N/V^350^I/K^351^E changes found in Tet(X3), they also contained some conservative amino acid substitutions hence they should be regarded as a group derived from further evolution events that occur among the TetX-B class variants. Functional classification is more informative than phylogenetic classification alone. In this work we found newly TetX-C class were distributed in three parts basing on phylogenetic analysis (Fig. 3). Sub-classification of each group is possible. For example, TetX-C enzymes can be sub-divided into TetX-(C1) to TetX-(Cn). If a new functional class of enzymes that contains a new set of conservative amino acid substitutions emerged, it can be classified as TetX-D. With the implementation of this new classification system, the nomenclature of TetX would be clear and in good order.

Due to the lack of clear classification of TetX, it has been difficult to investigate the evolutionary origin and bacterial host specificity of TetX variants. Using the newly developed classification system, we found that TetX-A clearly originated from Bacteroidetes, with *Bacteroides spp*., *Chryseobacterium spp*. and *Riemerella spp*. being the dominant host species (Table 3). Some TetX-A variants from *Riemerella spp*. further evolved into TetX-C. This theory is supported by the finding that the majority of the newly defined TetX-C variants are produced by strains of *Riemerella spp*. (83 out of 100 in *Bacteroidetes*, Table 3). Some mobile genetic elements such as plasmids carrying the TetX-C class variants were further disseminated to strains of Proteobacteria, which supported by three TetX-C class variants (WP_064754130.1, WP_044504094.1 and WP_117796890.1) harbored in both Bacteroidetes and Proteobacteria. Some TetX-A variants from *Empedobacter brevis* might have been evolved directly into the new TetX-B since TetX-B was only produced in *Empedobacter brevis* among species of *Bacteroidetes*, because the TetX-B variant (WP_150433336.1) and TetX-A variant (WP_150433355.1) were both from *Empedobacter brevis* strain SE1-3 but were located on different plasmid pSE1-3-9kb and pSE1-3-14kb, respectively (Table 3)^22^. This new *tet*X-C variant gene might be then transmitted to *Acinetobacter spp*. but mechanism underlying such transmission needs further investigation. Many species of *Proteobacteria* such as *E. coli*, *Salmonella spp*., *Klebsiella pneumoniae*, *Acinetobacter spp*., and *Pseudomonas spp*. are key bacterial pathogens that exhibit an increasing rate of drug resistance in recent years^24^. A high detection rate of tigecycline resistant strains that produce TetX variants means the effectiveness of tigecycline in treatment of bacterial infection would be compromised. With the emergence of mobile tigecycline resistance determinants in both zoonotic and clinical bacterial strains, as well as the continuous usage of tetracyclines in both clinical and non-clinical setting, the rate of resistance to tigecycline is expected to increase dramatically, diminishing its value as a last-resort antibiotic. Introducing a new classification system for tigecycline resistance determinants shall facilitate development of an effective molecular detection approach for more accurate assessment of the tigecycline susceptibility status of clinical strains and tracking the mobile resistance elements that they harbored, as well as design of proper antimicrobial regimen for treatment of infected patients.

## Methods

### Gene manipulation and mutagenesis

The *tet*(X2) (WP_063856436.1), *tet*(X3) (WP024160783), and *tet*(X4) (WP094309310) genes were amplified from dairy cows’ clinical isolates^25^ by PCR with primers listed in Supplementary Table 1. Then these genes were constructed into pBAD-18Kan vector and IPTG-inducible pET28b vector, respectively. The recombinant plasmids pBAD-18-*tet*(X2), pBAD-18-*tet*(X3) and pBAD-18-*tet*(X4) were transformed into *E. coli* BW25113 and followed by antimicrobial susceptibility tests. In addition, the recombinant plasmids pET28-6×His-*tet*(X2), pET28-6×His-*tet*(X3) and pET28-6×His-*tet*(X4) were transformed into *E. coli* strain BL21(DE3) for protein purification. Point mutations were introduced into the *tet*(X2) gene, *tet*(X3) gene and *tet*(X4) by using the QuickChange (Stratagene) commercial kit, following the manufacturer’s instructions, and confirmed by sequencing. Primers used in mutagenesis are also listed in Supplementary Table 1.

### Bioinformatics analysis

The sequence of a TetX variant (Accession number: WP_063856436.1) from *Bacteroides fragilis* is same to that of the first identified TetX (Q01911)^13,26,27^. PSI-BLAST (Position-Specific Iterated BLAST, accessed on 10 June 2021)^28^ was performed with the amino acid sequence of Tet(X2) (Accession number: WP_063856436.1)^23^ as the query sequence and searched on nr database with default value. The result yielded 128 TetX variants sequences with query cover >90% and percent identity >80%. These sequences were subjected to multiple sequence alignments by Clustal Omega^29^, the results were used to construct a phylogenetic tree using the online software PhyML 3.0^30^. The tree was visualized using iTOL^31^.

### Antimicrobial susceptibility tests

The MICs of three antibiotics (tetracycline, minocycline, and tigecycline) for strains were determined using the microbroth dilution method and the results were interpreted according to the CLSI guidelines^32^. For tigecycline, the breakpoint was interpreted according to the FDA criteria (susceptible, ≤2 mg/L; intermediate, 4 mg/L; resistant, ≥8 mg/L)^9^. *E. coli* strain ATCC 25922 was used as a quality control.

### Protein expression and Purification

Luria Broth (LB) containing 50 mg/L kanamycin was inoculated with 1% overnight culture, followed by incubation with shaking at 37 °C until an optical density of 0.6 at 600 nm (OD600) was reached. Expression of enzymes was induced by 0.5 mM IPTG at 16 °C for 16 h. The cells were harvested by centrifugation at 7000 rpm for 15 min and resuspended in lysis buffer (20 mM Tris-HCl, pH 8.0, 500 mM NaCl, 10 mM imidazole, and 1 mM protease inhibitor cocktails), and broken with sonication. The soluble fractions were passed through a Ni column, rinsed by 20 mM Tris-HCl, pH 8.0, 500 mM NaCl, and 10–30 mM imidazole, and finally eluted with 20 mM Tris-HCl, pH 8.0, 500 mM NaCl, and 250 mM imidazole. The 6xHis tag was removed by thrombin (Sigma, USA). The target proteins were further purified by gel filtration chromatography (Superdex 75; GE Healthcare) in a buffer of 20 mM Tris (pH 7.5), 150 mM NaCl, and 1 mM DTT. The desired fractions were collected and concentrated. The purity of protein was determined by SDS-PAGE (Supplementary Fig. 5).

### Steady-state kinetics of Tet(X) variants and mutants

Each enzyme reaction contains 0.1 M TAPS buffer at pH 8.5 with 0–40 μM substrate, 5 mM MgCl_2_ and 500 µM NADPH. Tin foil was used as light shield to protect substate and buffer. UV absorbance recorded in triplicate at 400 nm with a UV-1900 UV-Vis spectrophotometer (Shimadzu) for 3 minutes under a dim light condition at ambient temperature. Initial reaction velocities were determined for linear regression by the UVProbe 2.70 Software and fitted to the Michaelis–Menten equation using GraphPad Prism 8 (San Diego, CA, USA).

### Protein expression levels were measured by western blotting

Overnight cultures of *E. coli* BL21(DE3) carrying pET28-6 × His-*tet*(X2), pET28-6 × His-*tet*(X3), pET28-6 × His-*tet*(X4) and the variants were diluted 1:100 into 5 mL LB broth containing 50 μg/mL kanamycin. Cells were grown to OD_600_ = 0.6 at 37 °C and induced by adding 1 mM IPTG for 4 h at 30 °C. Cell was harvested by centrifugation at 13,000 rpm. Cell lysates were solubilized by boiling with SDS running buffer for 10 minutes and were subsequently separated by SDS-PAGE. Proteins were transferred to a PVDF membrane followed by blocking by skimmed milk for 1 h and incubated with mouse anti-6 × His antibody (ABCAM, USA) at 4 °C overnight. The goat anti-mouse antibody (ABCAM, USA) was used as the secondary antibody. The signal was generated by HRP substrate and detected by ChemiDoc Touch System (Bio-Rad, USA). Tet(X2) was used as a positive control on each protein gel and cells containing the empty vector was used as a negative control. The broad range anti-GADPH (ABCAM, USA) was used as loading control. Band intensities were quantified using ImageJ software.

### Protein structure analysis of TetX protein

The structure of Tet(X2) (PDB accession number 4A6N) was obtained from the Protein Data Bank (http://www.rcsb.org/pdb/). Structures of TetX variants were generated according to their amino acid sequence, using the comparative protein-modeling SWISS-MODEL server^33^. The structures were analyzed and showed by the PyMOL software^34^.

### Statistics and reproducibility

Statistical analysis was conducted with GraphPad Prism 8. Statistical methods used in this work are described in methods part and the figure legends.

## Supplementary information

Supplementary Information

## Data Availability

Plasmids harboring *Tet*(X2), *Tet*(X3), *Tet*(X4) are available on NCBI database as CP040909, CP041290 and, CP041286. Plasmid map of pET28b (#69865-3) and pBAD18-Kan are available on addgene. All other data are available from the corresponding author on reasonable request.
