# Supplementary material for: Functional and phylogenetic analysis of TetX variants to design a new classification system
Source: Commun Biol. 2022 May 31;5:522. doi: 10.1038/s42003-022-03465-y (PMC9156754; doi:10.1038/s42003-022-03465-y)
Supplement: Supplementary file 1 — Supplementary Information [file 42003_2022_3465_MOESM1_ESM.pdf]

Functional and phylogenetic analysis of TetX variants to design a new classification system

Qipeng Cheng[1, 2, #], Yanchu Cheung [2, #], Chenyu Liu[2], Edward Wai Chi Chan[1], Kwok Yin Wong[1], Rong Zhang[3], Sheng Chen[2]*

[1]State Key Lab of Chemical Biology and Drug Discovery, Department of Applied Biology and Chemical Technology, The Hong Kong Polytechnic University, Hung Hom, Kowloon, Hong Kong.

[2]Department of Infectious Diseases and Public Health, Jockey Club College of Veterinary Medicine and Life Sciences, City University of Hong Kong, Kowloon, Hong Kong.

[3]Department of Clinical Laboratory, Second Affiliated Hospital of Zhejiang University, School of Medicine, Hangzhou 310009, People's Republic of China

[#] These authors contributed equally.

*To whom correspondence should be addressed: Sheng CHEN, shechen@cityu.edu.hk

Keywords: TetX, Function, Evolution, Classification, Variants

Supplementary Table 1. Primers used in this study.

Primer	Sequence
tet(X2)-F[a]	CGCCATATGATGACAATGCGAATAGATACAG
tet(X2)-R[b]	CGCGGATCCTTATACATTTAACAATTGCTGAAAC
tet(X3)-R	CGCGGATCCTTATAGATTCAATAATTTTTGAAACGA
tet(X4)-F	CGCCATATGATGAGCAATAAAGAAAAACAAATG
H^{196}L-F	ATACAAGCCGATATTCTGCAACCAGAGATAAAC
H^{196}L-R	GTTTATCTCTGGTTGCAGAATATCGGCTTGTAT
Q^{197}H-F	CAAGCCGATATTCATCATCCAGAGATAAACTGT
Q^{197}H-R	ACAGTTTATCTCTGGATGATGAATATCGGCTTG
S^{217}A-F	AACCGGCTAATGGCAGCACACCAAGGTAATTTA
S^{217}A-R	TAAATTACCTTGGTGTGCTGCCATTAGCCGGTT
S^{217}G-F	AACCGGCTAATGGCAGGTCACCAAGGTAATTTA
S^{217}G-R	TAAATTACCTTGGTGACCTGCCATTAGCCGGTT
H^{279}R-F	TACAAAGAATTGATTCGTACGACGTTGTCATTT
H^{279}R-R	AAATGACAACGTCGTACGAATCAATTCTTTGTA
T^{280}S-F	AAAGAATTGATTCATAGCACGTTGTCATTTGTA
T^{280}S-R	TACAAATGACAACGTGCTATGAATCAATTCTTT
T^{280}V-F	AAAGAATTGATTCATGTGACGTTGTCATTTGTA
T^{280}V-R	TACAAATGACAACGTCACATGAATCAATTCTTT
T^{280}L-F	AAAGAATTGATTCATCTGACGTTGTCATTTGTA
T^{280}L-R	TACAAATGACAACGTCAGATGAATCAATTCTTT
L^{282}S-F	TTGATTCATACGACGAGCTCATTTGTAGGATTG
L^{282}S-R	CAATCCTACAAATGAGCTCGTCGTATGAATCAA
E^{295}N-F	CGGATATTTCCTTTAAACAAGCCTTGGAAAAGC
E^{295}N-R	GCTTTTCCAAGGCTTGTTTAAAGGAAATATCCG
E^{295}G-F	CGGATATTTCCTTTAGGAAAGCCTTGGAAAAGC
E^{295}G-R	GCTTTTCCAAGGCTTTCCTAAAGGAAATATCCG
E^{295}D-F	CGGATATTTCCTTTAGATAAGCCTTGGAAAAGC
E^{295}D-R	GCTTTTCCAAGGCTTATCTAAAGGAAATATCCG
K^{296}N-F	ATATTTCCTTTAGAAAACCCTTGGAAAAGCAAG
K^{296}N-R	CTTGCTTTTCCAAGGGTTTTCTAAAGGAAATAT
P^{297}D-F	TTTCCTTTAGAAAAGGATTGGAAAAGCAAGCGC
P^{297}D-R	GCGCTTGCTTTTCCAATCCTTTTCTAAAGGAAA
P^{297}S-F	TTTCCTTTAGAAAAGAGTTGGAAAAGCAAGCGC
P^{297}S-R	GCGCTTGCTTTTCCAACTCTTTTCTAAAGGAAA
V^{329}L-F	GTAAATAGTGGGTTGCTGGATGCCTTGATATTG
V^{329}L-R	CAATATCAAGGCATCCAGCAACCCACTATTTAC
V^{329}M-F	GTAAATAGTGGGTTGATGGATGCCTTGATATTG
V^{329}M-R	CAATATCAAGGCATCCATCAACCCACTATTTAC
A^{339}T-F	TTGTCTGATAATCTAACCGATGGAAAATTTAAT
A^{339}T-R	ATTAAATTTTCCATCGGTTAGATTATCAGACAA
D^{340}N-F	TCTGATAATCTAGCCAATGGAAAATTTAATAGC
D^{340}N-R	GCTATTAAATTTTCCATTGGCTAGATTATCAGA
V^{350}I-F	AGCATTGAAGAGGCTATTAAAAATTATGAACAG
V^{350}I-R	CTGTTCATAATTTTTAATAGCCTCTTCAATGCT
K^{351}E-F	ATTGAAGAGGCTGTTGAAAATTATGAACAGCAA
K^{351}E-R	TTGCTGTTCATAATTTTCAACAGCCTCTTCAAT
I^{359}V-F	GAACAGCAAATGTTTGTTTATGGCAAAGAAGCA
I^{359}V-R	TGCTTCTTTGCCATAAACAAACATTTGCTGTTC
I^{359}M-F	GAACAGCAAATGTTTATGTATGGCAAAGAAGCA
I^{359}M-R	TGCTTCTTTGCCATACATAAACATTTGCTGTTC
tet(X3)-L^{329}V-F	GTAAATACGGGATTAGTGGATGCTTTGATATTG
tet(X3)-L^{329}V-R	CAATATCAAAGCATCCACTAATCCCGTATTTAC

tet(X3)-$T^{339}A/N^{340}D$ -F	TTGTCTGAAAACCTTGCCGATGGAGAATTTACAAGT
tet(X3)-$T^{339}A/N^{340}D$ -R	ACTTGTAAATTCTCCATCGGCAAGGTTTTCAGACAA
tet(X3)-$I^{350}V/E^{351}K$ -F	AGTATTGAAAATGCCGTTAAAAACTACGAACAACAA
tet(X3)-$I^{350}V/E^{351}K$ -R	TTGTTGTTCGTAGTTTTTAACGGCATTTTCAATACT
tet(X4)-$M^{329}V$-F	GTAAACAGCGGGTTGGTGGATGCCTTGATATTG
tet(X4)-$M^{329}V$-R	CAATATCAAGGCATCCACCAACCCGCTGTTTAC
tet(X4)-$T^{339}A/N^{340}D$ -F	TTGTCGGATAATCTGGCCGATGGGAAATTTAACAGC
tet(X4)-$T^{339}A/N^{340}D$ -R	GCTGTTAAATTTCCCATCGGCCAGATTATCCGACAA
tet(X4)-$I^{350}V/E^{351}K$ -F	AGCATTGAAGAGGCTGTTAAAAATTATGAACAGCAA
tet(X4)-$I^{350}V/E^{351}K$ -R	TTGCTGTTCATAATTTTTAACAGCCTCTTCAATGCT

Note: [a] *tet*(X2)-F used for *tet*(X3) forward primer; [b] *tet*(X2)-R used for *tet*(X4) reverse primer.

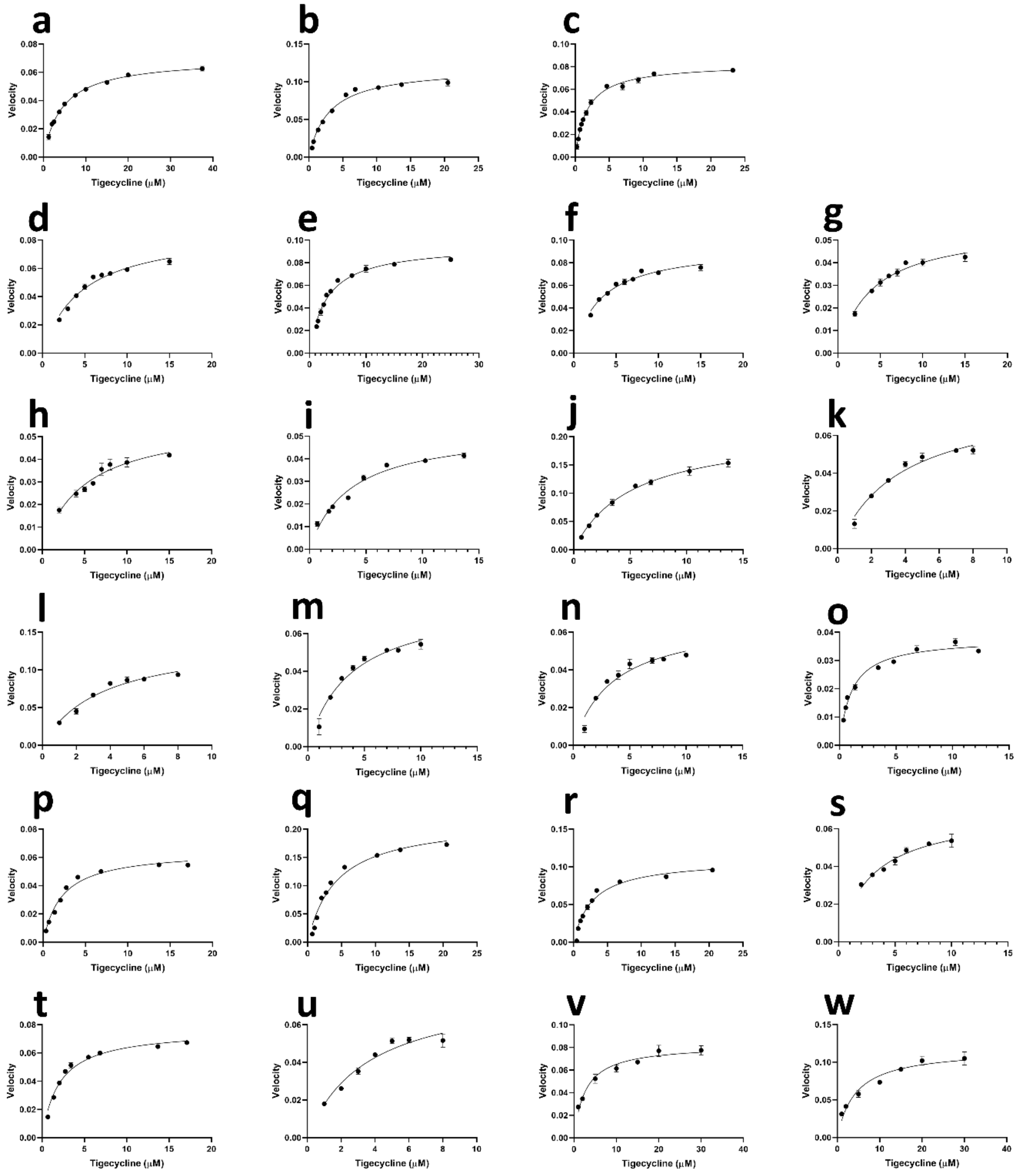

Supplementary Figure 1. Michaelis–Menten plot of Tet(X) variants and mutants. a Tet(X2), **b** Tet(X3),**c** Tet(X4), **d** S^{217}A, **e** T^{280}V, **f** T^{280}S, **g** L^{282}S, **h** E^{295}N, **i** V^{329}L, **j** V^{329}M, **k** A^{339}T, **l** K^{351}E, **m** A^{339}T/D^{340}N, **n** V^{350}K/I^{351}E, **o** V^{329}L/A^{339}T/D^{340}N, **p** V^{329}L/V^{350}I/K^{351}E, **q** V^{329}M/A^{339}T/D^{340}N, **r** V^{329}M/V^{350}I/K^{351}E, **s** A^{339}T/D^{340}N/V^{350}I/K^{351}E, **t** V^{329}L/A^{339}T/D^{340}N/V^{350}I/K^{351}E, **u** V^{329}M/A^{339}T/D^{340}N/V^{350}I/K^{351}E, **v** Tet(X3)-L^{329}V/T^{339}A/N^{340}D/I^{350}V/E^{351}K, **w** Tet(X4)-L^{329}V/T^{339}A/N^{340}D/I^{350}V/E^{351}K.

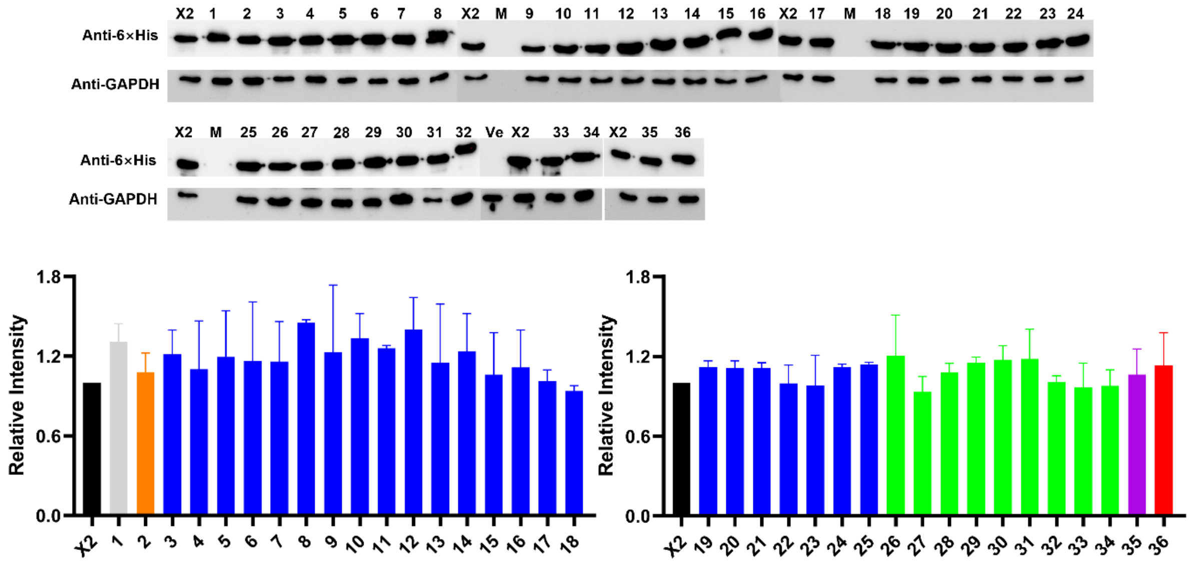

Supplementary Figure 2 Protein expression levels of TetX variant and mutant enzymes. **M** is the protein ladder, **Ve** is vector control, **X2** is Tet(X2) as a positive control and shown in black. **1** is Tet(X3) and shown in grey, **2** is Tet(X4) and shown in orange, **3** is H^{196}L, **4** is Q^{197}H, **5** is S^{217}A, **6** is S^{217}G, **7** is H^{279}R, **8** is T^{280}V, **9** is T^{280}L, **10** is T^{280}S, **11** is L^{282}S, **12** is E^{295}G, **13** is E^{295}N, **14** is E^{295}D, **15** is K^{296}N, **16** is P^{297}D, **17** is P^{297}S, **18** is I^{359}M, **19** is I^{359}V, **20** is V^{329}L, **21** is V^{329}M, **22** is A^{339}T, **23** is D^{340}N, **24** is V^{350}I, **25** is K^{351}E , **26** is A^{339T}/D^{340}N, **27** is V^{350I}/K^{351}E, **28** is V^{329}L/A^{339T}/D^{340}N, **29** is V^{329}L/V^{350I}/K^{351}E, **30** is V^{329}M/A^{339T}/D^{340}N, **31** is V^{329}M/V^{350I}/K^{351}E, **32** is A^{339T}/D^{340}N/V^{350I}/K^{351}E, **33** is V^{329}L/A^{339}T/D^{340}N/V^{350}I/K^{351}E, **34** is V^{329}M/A^{339}T/D^{340}N/V^{350}I/K^{351}E, **35** is Tet(X3)-L^{329}V/T^{339}A/N^{340}D/I^{350}V/E^{351}K and shown in purple, **36** is Tet(X4)-M^{329}V/T^{339}A/N^{340}D/I^{350}V/E^{351}K and shown in red. Single mutants are in blue and multiple mutants are in green. Band intensities from two independent experiments were used to plot the bar graph.

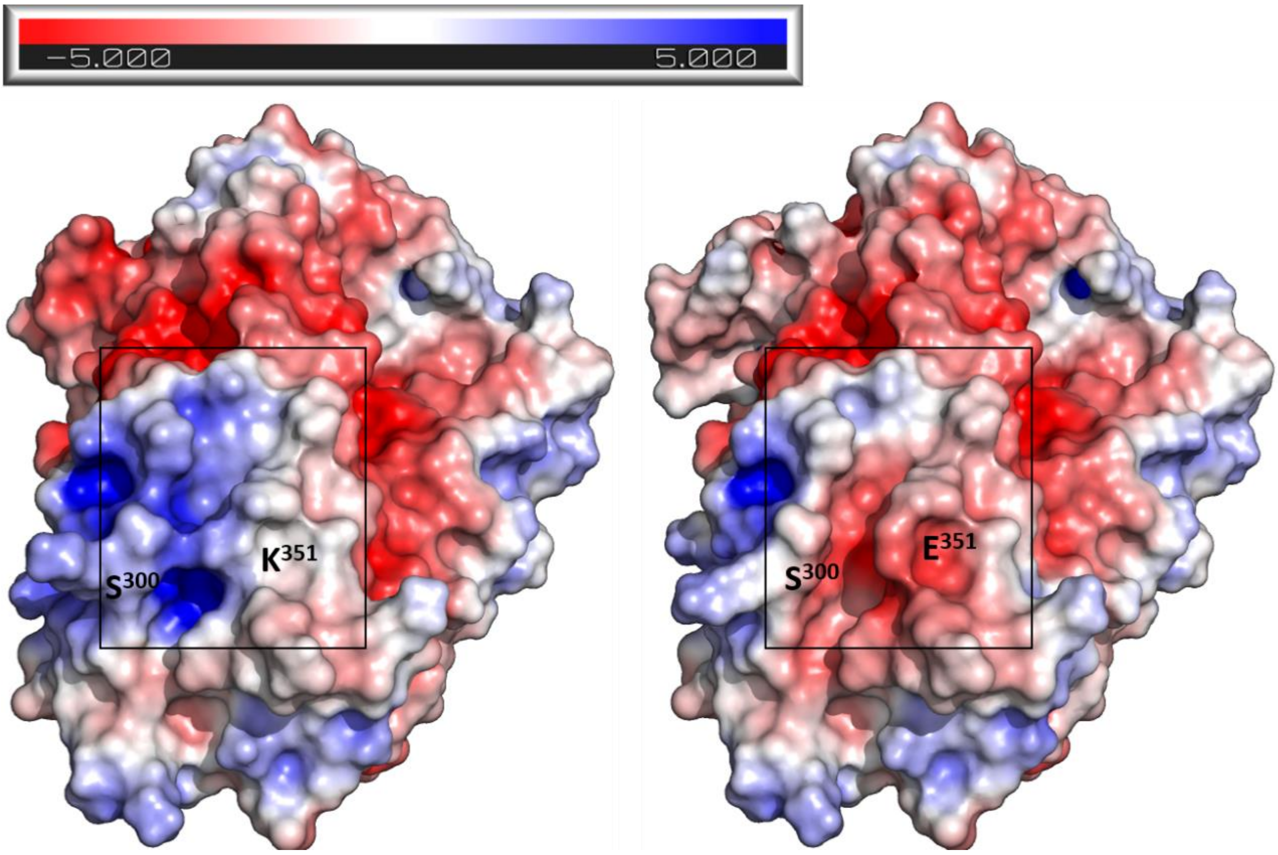

Supplementary Figure 3. Electrostatic potential map of Tet(X2) and the corresponsing mutants carrying the V^{329}M/A^{339}T/D^{340}N/V^{350}I/K^{351}E amino acid changes. The area between Loop 5 and C terminal α helix is marked by a black rectangle. The color change from red to blue reflects a shift of electrostatic potential from negative to positive.

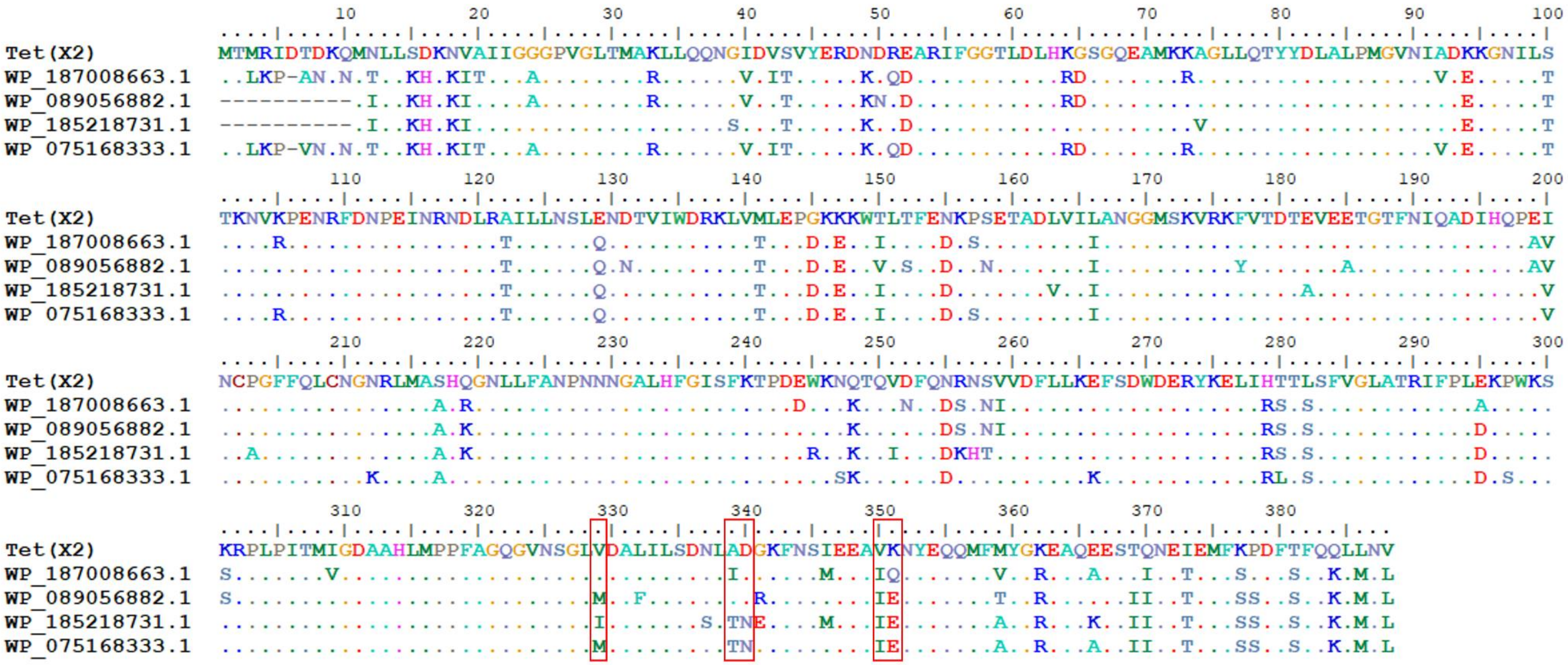

Supplementary Figure 4. Multiple alignment for WP_187008663.1, WP_089056882.1, and WP_185218731.1 with the variant (WP_075168333.1) of the Tet(X4) class in the same branch.

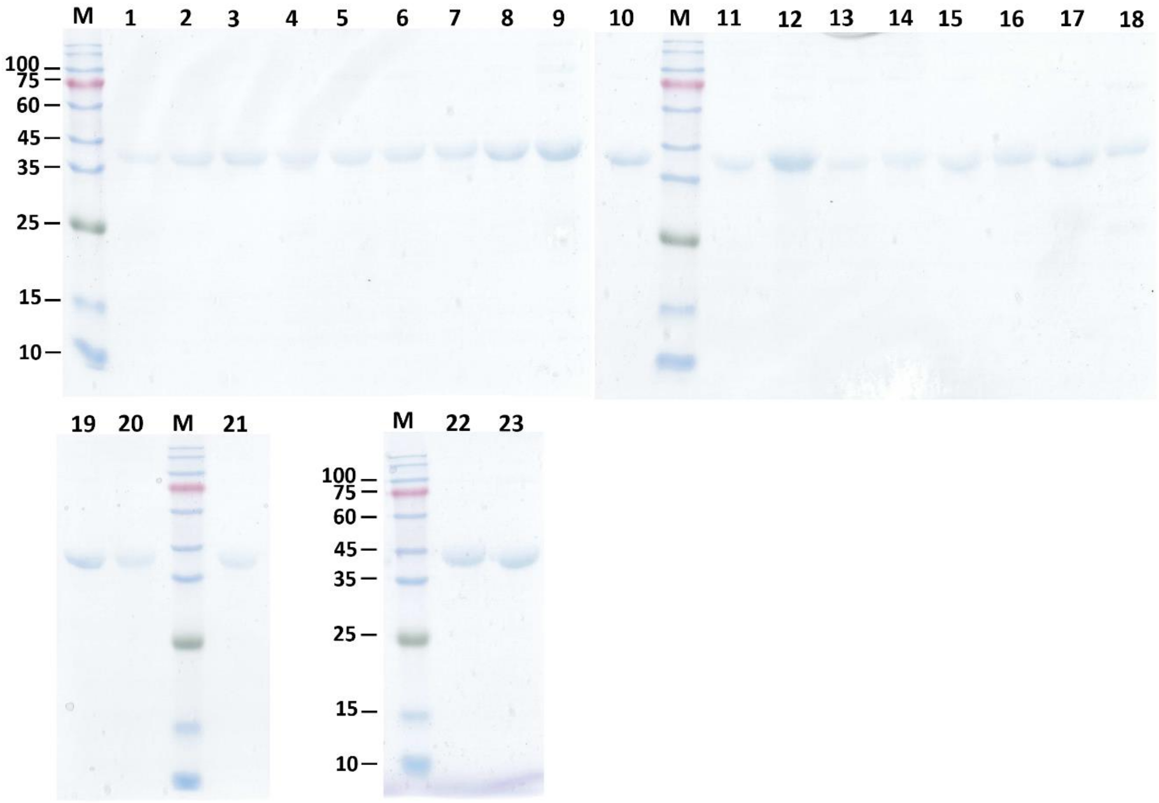

Supplementary Figure 5. SDS-PAGE analyses of recombinant TetX variants and mutants. **M** protein ladder**, 1** Tet(X2), **2** Tet(X3), **3** Tet(X4), **4** S^{217}A, **5** T^{280}V, **6** T^{280}S, **7** L^{282}S, **8** E^{295}N, **9** V^{329}L, **10** V^{329}M, **11** A^{339}T, **12** K^{351}E, **13** A^{339}T/D^{340}N, **14** V^{350}K/I^{351}E, **15** V^{329}L/A^{339}T/D^{340}N, **16** V^{329}L/V^{350}I/K^{351}E, **17** V^{329}M/A^{339}T/D^{340}N, **18** V^{329}M/V^{350}I/K^{351}E, **19** A^{339}T/D^{340}N/V^{350}I/K^{351}E, **20** V^{329}L/A^{339}T/D^{340}N/V^{350}I/K^{351}E, **21** V^{329}M/A^{339}T/D^{340}N/V^{350}I/K^{351}E, **22** Tet(X3)-L^{329}V/T^{339}A/N^{340}D/I^{350}V/E^{351}K, **23** Tet(X4)-L^{329}V/T^{339}A/N^{340}D/I^{350}V/E^{351}K.
